# Loss of Unilateral Lacrimation Following Adenoidectomy

**DOI:** 10.7759/cureus.9312

**Published:** 2020-07-21

**Authors:** Ayman A Mustafa, Michele M Carr

**Affiliations:** 1 Otolaryngology, Head and Neck Surgery, Jacobs School of Medicine and Biomedical Sciences, University at Buffalo, Buffalo, USA; 2 Otolaryngology, Jacobs School of Medicine and Biomedical Sciences, University at Buffalo, Buffalo, USA

**Keywords:** adenoidectomy, surgical complications, lacrimation, cranial nerves, pediatric

## Abstract

Complications following an adenoidectomy are rare. A 13-month-old female developed unilateral lacrimation impairment following an adenoidectomy and bilateral ventilation tube insertion. The patient’s post-operative course was marked by a fever, rhinorrhea, and dehydration. We suspect the impairment to be secondary to injury by suction cautery or post-operative inflammatory response and infection. Over the first nine months after surgery, the impairment spontaneously remitted.

## Introduction

An adenoidectomy is a common pediatric procedure that can be performed alone or in conjunction with ventilation tube insertion or tonsillectomy. The surgical procedure is frequently performed in children diagnosed with obstructive sleep apnea, chronic sinusitis that has been refractory to medical intervention, recurrent acute otitis media, or chronic otitis media with effusion. Complications following an adenoidectomy alone are rare and even more rarely life-threatening. Compared to a tonsillectomy or adenotonsillectomy, an adenoidectomy is associated with a markedly less morbidity [1]. Ventilation tubes are most often inserted due to recurrent otitis media with effusion, persistent middle ear fluid, or otitis media that persists following antibiotic therapy [2].

We describe the case of a 13-month-old female who had an adenoidectomy in conjunction with bilateral ventilation tube insertion and subsequently developed the inability to lacrimate from her left eye. The following case is distinctive as the post-operative lacrimation impairment observed is an unreported complication following adenoidectomy.

## Case presentation

A 13-month-old female presented to our clinic with rhinorrhea, diarrhea, and an inability to tear from her left eye at 21 days status post-adenoidectomy and bilateral ventilation tube insertion, which was performed at another institution. Presenting complaints also included weight loss, oral fetor, and poor oral intake. Her parents noted that she was squinting more than usual, and frequently rubbing her left eye. This behavioral change was noticed more than two weeks following surgery.

Her past history included an unremarkable delivery at 37 weeks gestation, as well as an early diagnosis of supraventricular tachycardia, which was treated with digoxin in the perinatal period. She was otherwise healthy with no known allergies and fair growth noted prior to surgery.

**Figure 1:**
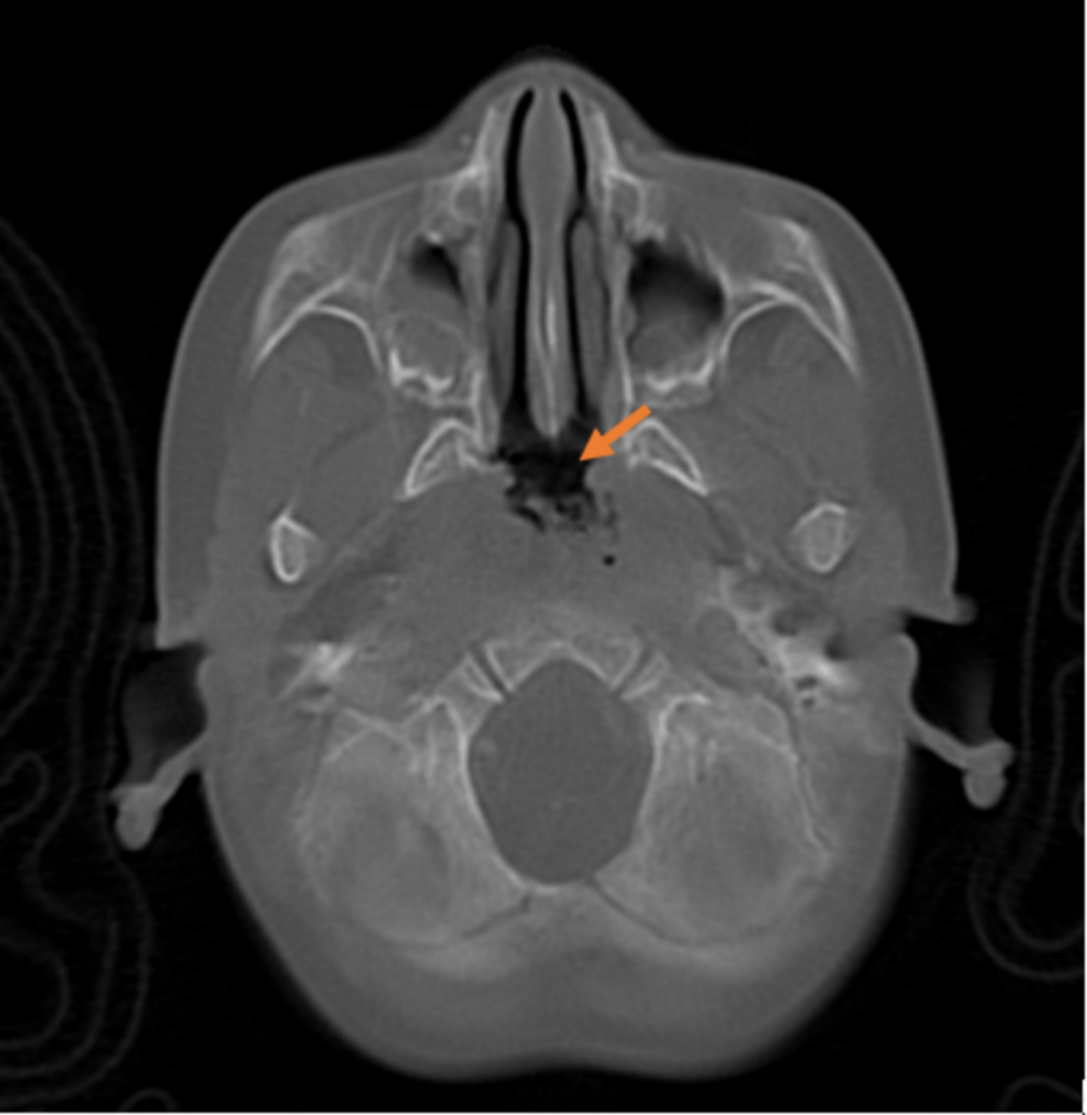
CT scan of nasopharynx two weeks following adenoidectomy Mild edema and mucus observed within the nasopharyngeal area (orange arrow). Additional views revealed normal bone in the skull base.

Her pre-operative diagnosis had been persisting middle ear effusions and obstructive sleep apnea. She had a suction cautery adenoidectomy according to operative notes. On her first post-operative night, the patient had oxygen desaturations and received intravenous steroids. Following her discharge from the hospital, she had a low-grade fever which led to her parents taking her to the emergency department on two different occasions. She was treated with antibiotics including amoxicillin-clavulanate and ceftriaxone. The patient was admitted to our institution on post-operative day 13 with persisting fever and dehydration. A computed tomography (CT) scan of her sinuses and skull base was obtained, revealing clear sinuses with no evidence of osteomyelitis of the skull base (Figure 1). Physical exam revealed ventilation tubes in each tympanic membrane which appeared clean, dry, and open. The left side of her nasal cavity was clear while the right side was occluded with mucus. An erythematous pharynx was observed. She had full range of neck mobility with no masses palpable. When she cried, no tears were observed from the left eye. The sclerae were clear and pupils reactive to light. She was diagnosed with surgical site infection and discharged with a continued course of antibiotics.

On post-operative day 28, the patient’s rhinorrhea improved, her diarrhea resolved, and her feeding was improving. Physical examination revealed a clear oral cavity and pharynx. Her right nasal cavity was clear and the left nasal cavity showed some crusts of dry mucus. Several small cervical lymph nodes were palpable bilaterally. A flexible laryngoscopy was performed which revealed a thick mucus coating the site of the adenoidectomy procedure.

On post-operative day 29, the patient was evaluated by a pediatric ophthalmologist who noted an objective difference in tear production via a Schirmer test and that otherwise, her eyes were normal. No special care was recommended.

On post-operative day 60 the patient’s rhinorrhea, poor feeding, and irritability had completely resolved. Lacrimation remained asymmetrical with the left eye lacking tear production. Physical examination revealed clean, dry, and open ventilation tubes in each of her tympanic membranes. The patient’s nose was clear and her nasal airway wide open. The remainder of the physical examination was noncontributory. Follow-up telephone contact with the family six months following this encounter revealed that the family noted that tearing had returned to normal.

## Discussion

Following an adenoidectomy, a patient may typically experience a sore throat, neck pain, or dysphagia. Rare but significant complications of an adenoidectomy include post-operative hemorrhage, velopharyngeal insufficiency, torticollis, nasopharyngeal stenosis, atlantoaxial subluxation (Grisel Syndrome), as well as cervical or skull base osteomyelitis [3-6]. If removal of the adenoid is incomplete, it may regrow [7]. A mouth gag is utilized for the duration of the procedure to ensure the patient’s mouth remains open. Rarely, use of this medical equipment may cause dysfunction of the temporomandibular joint [8].

This case describes spontaneously remitting unilateral lacrimation impairment following an adenoidectomy in a 13-month-old child. Tear production occurs at the lacrimal and accessory lacrimal glands in response to emotional distress or noxious stimuli. The lacrimal glands are located on the upper lateral portion of the orbit, while accessory lacrimal glands are located along the conjunctiva. The afferent pathway of the lacrimation reflex arc is the ophthalmic branch of the trigeminal nerve. The efferent pathway consists of both parasympathetic and sympathetic fiber contribution.

**Figure 2:**
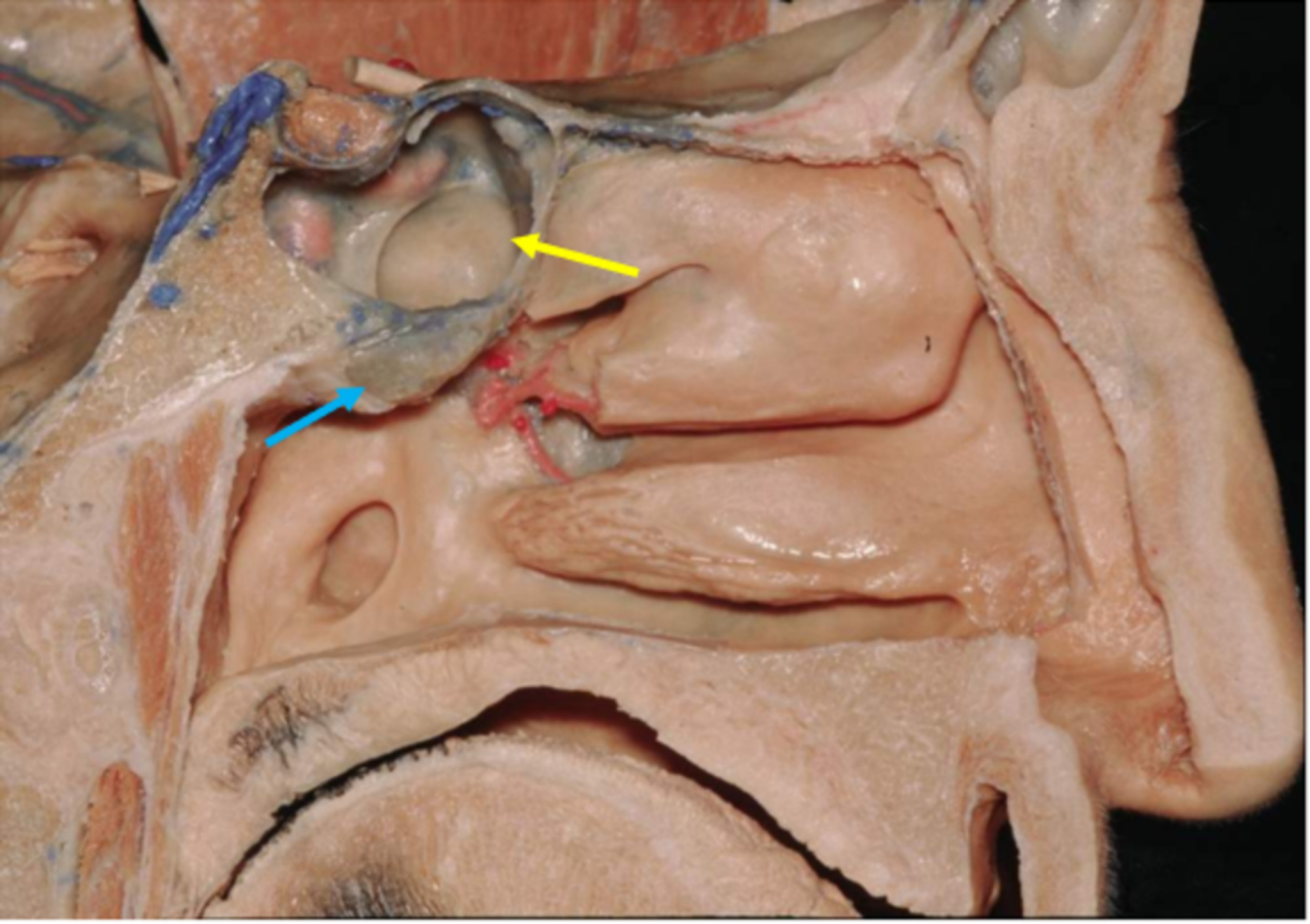
Sagittal view of left nasal cavity Adenoid (blue arrow) located inferior to sphenoid sinus (yellow arrow). Reproduced with permission from The Rhoton Collection.

**Figure 3:**
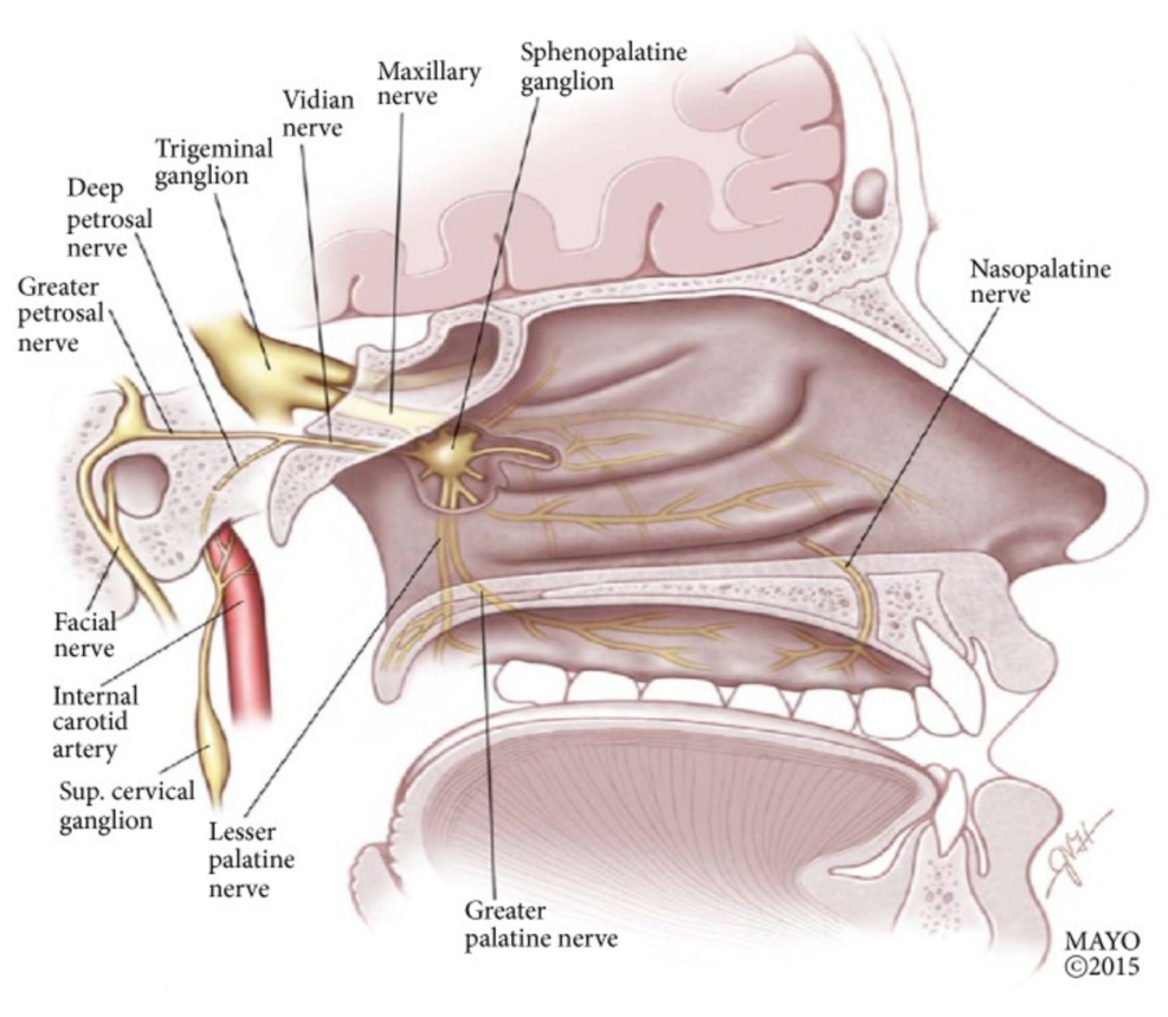
Sagittal view of the nasopharynx, demonstrating the sphenopalatine ganglion and its neural connections Reproduced with permission from Robbins et al. (2016) [under the Creative Commons Attribution License number 4844340309891 (Wiley)].

Parasympathetic innervation of the lacrimal apparatus originates from the superior salivatory nucleus in the pontine tegmentum and joins both general somatic sensory and special sensory fibers to form the nervus intermedius. The preganglionic parasympathetic fibers enter the geniculate ganglion, continuing their course without synapsing in the ganglion and exits as the greater petrosal nerve. At the geniculate ganglion, the nervus intermedius splits into the greater petrosal nerve which innervates the lacrimal glands, and the chorda tympani. The greater petrosal nerve exits the petrous portion of the temporal bone via the hiatus for the greater petrosal nerve to enter the middle cranial fossa. The nerve passes deep to the trigeminal ganglion to the foramen lacerum, through which it travels to the pterygoid canal. In the pterygoid canal, the greater petrosal nerve joins the deep petrosal nerve to form the nerve of the pterygoid canal, also known as the Vidian nerve. Axons from the Vidian nerve will synapse in the sphenopalatine ganglion; postganglionic parasympathetic fibers are carried via branches of the maxillary division of the trigeminal nerve to the lacrimal gland [9-11]. The adenoid is located cephalad and inferior to the sphenoid sinus (Figure 2) [12]. The sphenopalatine ganglion would be found lateral and anterior to the adenoid (Figure 3) [13].

A potential mechanism for the unilateral lacrimation impairment observed in our patient may involve neural injury inflicted by the suction cautery employed during adenoidectomy. Temperatures exceeding 60 °C are sufficient to result in thermal soft tissue damage, which occurs at a suction cautery setting of 40 watts [14]. High wattage from the suction cautery may have resulted in damage to the nasal wall leading to injury of the nearby sphenopalatine ganglion or Vidian nerve. This scenario would imply an intraoperative onset of injury and that the impairment went unnoticed for the initial two weeks following the procedure. Notes regarding the intraoperative events were not obtainable to gain additional insight into the procedure performed. Excessively high wattage set on the suction cautery, presence of adenoid tissue within the choanae, or difficulty visualizing the operative site would further support an intraoperative cause of the patient’s impairment.

A true delay in onset of the impairment favors an inflammatory etiology. This would suggest that the subsequent infection at the surgical site resulted in neural injury. A substantial increase in proinflammatory cytokines including interleukin (IL)-1, IL-2, IL-6, and tumor necrosis factor α (TNF-α) is commonly observed following elective or urgent surgery or significant trauma [15-16]. The presence of an infection would further exacerbate and prolong the inflammatory response [15]. The proinflammatory cascade from the typical postoperative inflammation and surgical site infection results in macrophage and leukocyte recruitment to the site of injury in conjunction with increased vascular permeability [15]. This increased permeability results in a substantial perineural inflammatory infiltration. Segmental demyelination and axonal degeneration of nerve fibers have been shown to occur in the vicinity of infiltrating mononuclear cells [17-18]. These pathologic neural injury mechanisms have been strongly associated with the activation of macrophages [18]. The proteases and urokinase involved in the activation of macrophages also have the potential to act as potent demyelinating agents of neighboring nerve fibers [18]. It has been proposed that urokinase, a known plasminogen activator released by the stimulated macrophages, results in the generation of plasmin which then hydrolyzes the basic protein in intact myelin [19]. This structural alteration has a drastic negative effect on impulse propagation throughout the nerve affected, as demyelination directly affects conduction and also results in changes in both the distribution and function of expressed axolemmal ion channels [20]. Function is deeply influenced by structure both from a molecular and gross anatomical perspective.

## Conclusions

This case demonstrates a previously unreported complication following adenoidectomy, namely a transient unilateral absence of tear production. We suggest possible mechanisms although it is possible that a separate, unidentified, temporally coincident process accounted for this event. Despite this possibility, and the fact that adenoidectomy has a low incidence of complication, surgeons should be cognizant of the sphenopalatine ganglion’s proximity to the operative site.
